# Endothelial cell‐derived matrix promotes the metabolic functional maturation of hepatocyte *via* integrin‐Src signalling

**DOI:** 10.1111/jcmm.13195

**Published:** 2017-05-04

**Authors:** Xinyue Guo, Weihong Li, Minghui Ma, Xin Lu, Haiyan Zhang

**Affiliations:** ^1^ Department of Cell Biology Municipal Laboratory for Liver Protection and Regulation of Regeneration Capital Medical University Beijing China

**Keywords:** endothelial cells, extracellular matrix, hepatocyte, metabolism, integrin, Src

## Abstract

The extracellular matrix (ECM) microenvironment is involved in the regulation of hepatocyte phenotype and function. Recently, the cell‐derived extracellular matrix has been proposed to represent the bioactive and biocompatible materials of the native ECM. Here, we show that the endothelial cell‐derived matrix (EC matrix) promotes the metabolic maturation of human adipose stem cell‐derived hepatocyte‐like cells (hASC‐HLCs) through the activation of the transcription factor forkhead box protein A2 (FOXA2) and the nuclear receptors hepatocyte nuclear factor 4 alpha (HNF4α) and pregnane X receptor (PXR). Reducing the fibronectin content in the EC matrix or silencing the expression of α5 integrin in the hASC‐HLCs inhibited the effect of the EC matrix on Src phosphorylation and hepatocyte maturation. The inhibition of Src phosphorylation using the inhibitor PP2 or silencing the expression of Src in hASC‐HLCs also attenuated the up‐regulation of the metabolic function of hASC‐HLCs in a nuclear receptor‐dependent manner. These data elucidate integrin‐Src signalling linking the extrinsic EC matrix signals and metabolic functional maturation of hepatocyte. This study provides a model for studying the interaction between hepatocytes and non‐parenchymal cell‐derived matrix.

## Introduction

The severe shortage of liver donors for human primary adult hepatocyte has prompted *in vitro* efforts to produce the functional hepatocyte‐like cells (HLCs) from stem cells 1, 2. Human adipose stem cells (hASCs) have the same genetic makeup as the donor patient, making them an ideal cell source for disease modelling, hepatotoxicity testing, and cell‐based therapies 3, 4, 5. As a result, HLCs have been differentiated from hASCs *in vitro* by toning soluble maintenance factors or microRNA to direct cell fate 5, 6, 7, 8. However, the ability of stem cell‐derived HLCs to replicate the function of endogenous cells is limited by a blunted phenotype with reduced metabolic enzyme activity 9, 10, 11. The key challenge is to recreate cell–their surrounding extracellular matrix (ECM) interactions, that is *in vivo* liver microenvironment, which plays a vital role during liver development, and hepatocyte functional maturity 4, 12, 13. The ECM proteins produced provide not only an adhesive substrate for integrins but also a platform for the transduction of intracellular signalling events that regulate a host of cell functions and that play an important role in maintaining the differentiated phenotype 12, 14, 15.

Recent studies have suggested that combinatorial biochemical ECM signalling 16 and acellular tissue‐derived matrix 4, 17 are more effective than traditional collagen sandwich cultures to functional maturation of HLCs. Considering the complexity of the ECM *in vivo* environment, it is difficult to elucidate the comprehensive roles of the naive ECM in hepatocyte differentiation using a mixture of single ECM molecules. In contrast to tissue‐derived matrix, cell‐derived matrix contains a complex yet organized mixture of macromolecules that includes bioactive and biocompatible materials, which together represent, at least to some extent, the composition and organization of native ECM 18, 19, 20, 21. In addition, the cell‐derived matrix has a greater ability for customization because the type(s) of cells used can be selected to generate the ECM according to the relevant *in vivo* environment 22.

As one of the major cellular components of liver, endothelial cells act as a cellular node that guides the essential steps in liver formation 23 and that governs the regeneration, homeostasis and pathology of the liver 24. When co‐cultured with hepatocytes *in vitro*, endothelial cells may improve and maintain the function of primary hepatocytes 25, 26, 27, and they can mediate hepatocyte recruitment in the establishment of liver‐like tissue 28. Although the angiocrine soluble signals that mastermind these complex tasks have been partially defined 24, the contribution of the endothelial cell produced matrix has not been well studied until now. The matrix derived from endothelial cells has been used to study adhesion‐ and growth‐promoting properties of vascular endothelial cells and to assess endothelial differentiation potential from human mesenchymal stem cells 22, 29, 30. Fibronectin (FN) and collagen I are the main components of the ECM produced by cultured endothelial cells 18, 30, and the properties of this ECM closely resemble the properties of the ECM located in the space of Disse in liver 12, which separates the sinusoidal endothelium from the underlying hepatocytes. We wondered whether endothelial cell‐derived matrix (EC matrix) could regulate the phenotype and function of hepatocytes.

In this study, the EC matrix promoted the metabolic functional maturation of hASC‐derived HLCs (hASC‐HLCs) by regulating the activity of the transcription FOXA2 and the nuclear receptors HNF4α and PXR in an α5β1 integrin‐ and Src‐dependent manner.

## Materials and methods

### Human volunteers

All experimental protocols involving human tissues and cells were reviewed, approved and carried out in accordance with the relevant guidelines and regulations of the Ethics Committee of Capital Medical University.

### Preparation of coverslips coated with different types of matrix

To coat coverslips with different types of matrix, the glass coverslips (NEST Biotechnology, Wuxi, China) were placed into 24‐well cell culture plates (Sigma‐Aldrich, St. Louis, MO, USA); 500 μl of a solution of 2 mg/ml gelatin (Sigma‐Aldrich) was applied to each well. After one‐hour incubation at 37°C, the coverslips were washed with phosphate‐buffered saline (PBS) and then exposed for 30 min. to phosphate‐buffered solution (PB) containing 1% glutaraldehyde at room temperature. The coverslips were washed with PBS, then treated for 30 min. with PBS containing 1 M ethanolamine at room temperature and then washed three times extensively with PBS.

To prepare the EC matrix, primary human umbilical vein endothelial cells (HUVECs) were isolated using 0.2 mg/ml collagenase II (Sigma‐Aldrich) and cultured in endothelial cell growth medium (Promo Cell GmbH, Heidelberg, Germany). Cells from passages 1 to 3 were used in this study. HUVECs were seeded on the gelatin‐treated coverslips as described above at a density of 52,000/cm^2^. Once became confluent (approximately 72 hrs), the cells were washed with PBS and exposed for 5 min. to PBS containing 0.3% (vol/vol) Triton X‐100 and 20 mM NH_4_OH at 37°C. The cultures were washed three times with PBS. After washing, the properties of the resulting surface material were examined by scanning electron microscopy (SEM) and immunofluorescence, and then referred to here as EC matrix as previously 20. EC matrix was immediately used or stored at 4°C for up to 2 weeks.

To coat with FN, the gelatin‐treated coverslips were placed into 24‐well cell culture plates; 500 μl of a solution of 3 ug/ml human FN (Sigma‐Aldrich) in Hank's medium (Invitrogen, Grand Island, NY, USA) was added to the wells. After an incubation period of 2 hrs at 37°C, the coverslips were washed three times with PBS. To coat with collagen I, the gelatin‐treated coverslips were incubated with 500 μl of a solution of 0.5 mg/ml rat tail collagen I (BD Biosciences, San Jose, CA, USA) for 60 min. at 37°C and washed extensively with PBS. To coat with collagen I plus FN, the gelatin‐treated coverslips were coated with collagen I firstly; then, 500 μl of a solution of 3 ug/ml human FN (Sigma‐Aldrich) in Hank's medium (Invitrogen) was added to the wells. After an incubation period of 2 hrs at 37°C, the coverslips were washed three times with PBS.

### Cell culture, hepatic differentiation and cell seeding

hASCs from four different donors were separately cultured and differentiated to HLCs as previously described 7. Briefly, hASCs were plated on collagen I‐coated dishes and cultured in DMEM/F‐12 (Invitrogen) supplemented with 10% foetal bovine serum mesenchymal stem cell screened (FBS‐MSCS, HyClone, Logan, UT, USA), 100 U/ml penicillin and 100 μg/ml streptomycin (Invitrogen) at 37°C with 5% CO2. Once the cells reached 90% confluence, they were washed twice with PBS and incubated with serum‐free DMEM/F‐12 medium for 48 hrs. The cells were incubated with DMEM/F‐12 containing 0.5 mg/ml albumin fraction V (BSA; Sigma‐Aldrich) and 100 ng/ml activin A (Peprotech, Rocky Hill, NJ, USA) for 72 hrs; 1% insulin–transferrin–selenium (ITS) (Sigma‐Aldrich) was added to the medium beginning at the second day. At day 5, the medium was changed to MEM/NEAA (Invitrogen), supplemented with 0.5 mg/ml BSA, 1% ITS, 20 ng/ml bone morphogenetic protein 2 (BMP2) (Peprotech) and 30 ng/ml fibroblast growth factor 4 (FGF4) (Peprotech) for 5 days. At day 10, the cells were further treated with 20 ng/ml hepatocyte growth factor (HGF; Peprotech) for 5 days and with 20 ng/ml HGF, 10 ng/ml oncostatin M (OSM; Peprotech) plus 10^−6^M dexamethasone (DEX; Sigma‐Aldrich) treatment for another 5 days. The differentiation media were changed every 2 days.

Once the hASC‐HLCs were differentiated, these cells were harvested and seeded on either EC matrix, collagen I, FN or collagen I plus FN‐coated substrate separately at a density of 75,000/cm^2^ with MEM/NEAA, supplemented with 0.5 mg/ml BSA, 1% ITS, 20 ng/ml HGF, 10 ng/ml OSM plus 10^−6^M DEX. After a 24‐hr incubation, the medium were changed to hepatocyte maintenance medium (PromoCell GmbH, Heidelberg, Germany). Following culture on different substrates, the metabolic properties of the hASC‐HLCs were analysed further.

Human hepatocytes (ScienCell Research Laboratories, Carlsbad, CA, USA) from three different batches were cultured on EC matrix or collagen I‐coated substrate separately at a density of 75,000 cells/cm^2^ with hepatocyte medium (ScienCell Research Laboratories). After 24‐hr incubation, the medium was changed to hepatocyte maintenance medium (PromoCell).

### Effect of inhibitors

hASC‐HLCs were seeded on EC matrix at a density of 75,000/cm^2^. After a 6‐hr incubation, focal adhesion kinase (FAK) inhibitor (1 μM PF228; Selleckchem, Houston, TX, USA), Src family kinase (SFK) inhibitor (5 μM, PP2, Selleckchem) and integrin‐linked kinase (ILK) inhibitor (1 μM Cpd22, Merck Millipore, Darmstadt, Germany) were added separately for 15 hrs; then, the cells were cultured for 72 hrs, and the metabolic properties of the hASC‐HLCs were analysed further.

### siRNA transfection

hASC‐HLCs or HUVECs were plated in antibiotic‐free basal medium 24 hrs prior to transfection. siRNA transfection was performed following the manufacturer's protocol as previously described. Briefly, ON‐TARGET SMARTpool siRNAs directed against human α*5 integrin* (L‐008003‐00‐0005, Dharmacon, Lafayette, LA, USA), human *FN* 1 (L‐009853‐00‐0005, Dharmacon), human *Src* (L‐003175‐00‐0005, Dharmacon) or non‐targeting siRNAs (D‐001810‐10‐05, Dharmacon) were mixed with Transfection DharmaFECT 4 (Dharmacon) or Transfection DharmaFECT 1 (Dharmacon). After a 20‐min. incubation at room temperature, the complexes were added to the cells at a final siRNA concentration of 50 nM. The medium was replenished with medium containing antibiotics for 24 hrs after transfection. The culture medium was changed every 2 days for the duration of the experiment.

### Immunofluorescence

Immunofluorescence analysis was performed as previously described 7. Briefly, the cells or EC matrix was fixed with 4% paraformaldehyde for 20 min. at room temperature, followed by permeabilization with 0.3% Triton X‐100 in PBS for 5 min. The cells were rinsed and blocked with either 10% goat serum (Zsgb‐Bio, Beijing, China) or 5% BSA (Invitrogen) for 60 min. at room temperature. The cells were then incubated with the primary antibodies, which are listed in Table S1, at 4°C overnight. Following three 5‐min. washes in PBS with gentle agitation, an Alexa Fluor‐conjugated secondary antibody (Invitrogen) at 1:500 was added, and the samples were incubated for 1 hr at 37°C. The nuclei were counterstained with 4′,6‐diamidino‐2‐phenylindole (DAPI, Sigma‐Aldrich).

### Image acquisition and analysis

Immunofluorescence images of the stained cells were examined and acquired under a Leica TCS SP8 confocal microscope (Leica, Wetzlar, Germany). For DPP4 density measurements in the cells, 10 fluorescence images from each coverslip were randomly captured using Leica TCS SP8 confocal microscope equipped with HC PL Apo×63/1.4 oil immersion CS2 objective. Immunofluorescence density analysis was performed using Image J software (National Institutes of Health, Bethesda, MD, USA). The relative expression of protein was presented with fold over the control.

### SEM

SEM analysis was performed as previously described 4. The samples were examined using a Hitachi S‐4800 scanning electron microscope (Hitachi, Tokyo, Japan).

### Flow cytometry

For the flow cytometric detection of α5, β1 and α5β1 integrin, the cells were fixed using fixation/permeabilization working solution (Affymetrix eBioscience, Inc., San Diego, CA, USA) for 60 min. After being washed twice, the cells were blocked with 2% normal goat serum for 60 min. at room temperature; then, relative antibodies (Table S1) were added and incubated at room temperature for 60 min. Following two 5‐min. washes with gentle agitation, an Alexa Fluor‐conjugated secondary antibody at 1:500 (Invitrogen) was added, and the samples were incubated for 60 min. at room temperature. Following two 5‐min. washes, the cell suspensions were resuspended in permeabilization working solution, and flow cytometry (BD Accuri C6; BD Biosciences) was carried out. Unstained control and isotype control cells were included in each experiment to obtain threshold setting. FACS profiles were produced using FLOWJO™ software (TreeStar Inc., Ashland, OR, USA).

### Real‐time RT‐PCR

Real‐time RT‐PCR was performed as previously described 31, 32. Total cellular RNA was extracted from 2.5 × 10^5^ cells with the RNeasy Mini Kit (Qiagen, Hilden, Germany) according to the manufacturer's instructions. For PCR analysis, 1 μg of RNA was reverse‐transcribed to cDNA using Superscript III reverse transcriptase and random hexamer primers (Invitrogen). Real‐time PCR analysis was performed on an ABI Prism 7300 Sequence Detection System using the SYBR Green PCR Master Mix (Applied Biosystems, Foster City, CA, USA). The reaction mixture consisted of 10 μl of SYBR Green PCR Master Mix, 1 μl of a 5 μM mix of forward and reverse primers, 8 μl of water and 1 μl of template cDNA in a total volume of 20 μl. Cycling was performed using the default conditions of ABI 7300 SDS Software 1.3.1. The primers used are listed in Table S2. The relative expression of each gene was normalized against 18S rRNA. Relative mRNA expression was presented with fold over the control 33. The data are presented as the mean ± S.D.

### Western blot

Adherent cells were lysed on ice using Nuclear and Cytoplasmic Extraction Reagent Kit (NE‐PER) (Thermo Scientific, Waltham, MA, USA). Samples were normalized for protein concentration using the BCA Protein Assay. Each sample (15–20 μg) was analysed by 10% SDS‐PAGE (Invitrogen) and transferred to a PVDF membrane (Merck Millipore). Membranes were blocked in 5% BSA in TBS and incubated overnight at 4°C with the specific primary antibodies (Table S1). Glyceraldehyde phosphate dehydrogenase (GAPDH) was used as internal reference. The membranes were washed with TBS and incubated with IRDye‐conjugated secondary antibodies (Table S1) for 1 hr at room temperature. The membranes were scanned with the Odyssey detection system (Li‐COR, Lincoln, NE, USA). Relative densities of proteins were quantitatively assayed using Image J. Relative mRNA expression was presented with fold over the control. The data are presented as the mean ± S.D.

### CYP450 activity assay

CYP2C9 and CYP3A4 enzyme activities were assessed by P450‐Glo assay (Promega, Madison, WI, USA), according to the manufacturer's instructions. Differentiated cells were treated with or without 25 μM rifampicin (Sigma‐Aldrich), a PXR agonist, for 72 hrs, and the media were changed on the cells every day. The cells were incubated at 37°C in fresh medium with Luciferin‐H for 4 hrs or Luciferin‐IPA for 60 min. separately. After the incubation, 50 μl of medium was transferred to a 96‐well plate and mixed with 50 μl of luciferin detection reagent to initiate the luminescent reaction. After 60 min. of incubation at 37°C, the luminescence was measured with a luminometer (Envision2104‐0010, Waltham, MA, USA).

### Statistical analysis

At least three independent determinations of each parameter were compared among the treatment groups by one‐way anova using the statistical software SPSS 11.5 (IBM Corporation, Armonk, NY, USA). Differences were considered significant if *P* < 0.05.

## Results

### Properties of the EC matrix

To characterize the ECM secreted by endothelial cells *in vitro*, the expression of ECM proteins in HUVECs was determined. The results showed that the major components of the ECM, such as FN, collagen I, collagen IV and laminin (LN), were identified in cultured HUVECs (Fig. S1). Immunofluorescence staining showed that FN, collagen I and collagen IV were located both in and under the cells. FN appeared as abundant fibrils distributed around the cells. In contrast, LN was located in the cells (Fig. S1).

**Figure 1:**
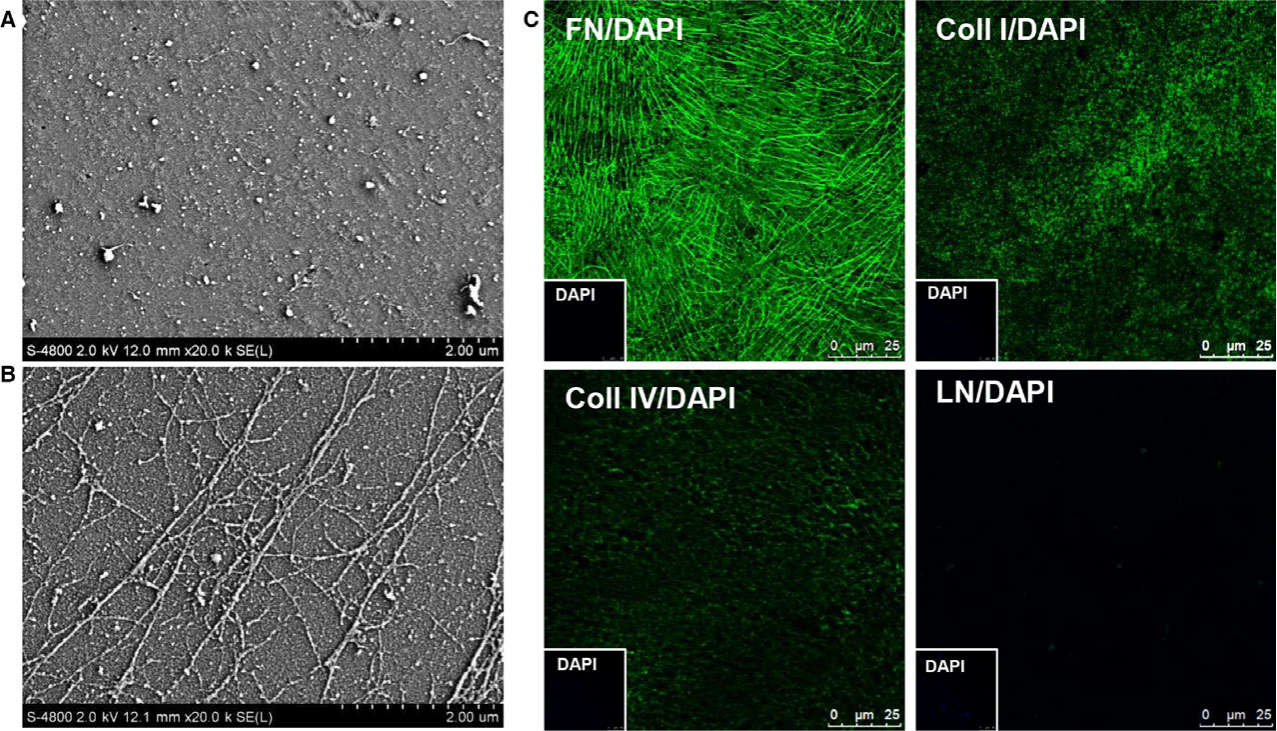
Characterization of the matrix. Surface views of the collagen I‐coated substrate (**A**) and the EC matrix (**B**) were examined using SEM Scale bars, 2 μm. (**C**) Immunofluorescence staining for FN, collagen I, collagen IV and LN in the EC matrix. Insets: The EC matrix was counterstained with DAPI. Scale bars, 25 μm. Coll I, collagen I; Coll IV, collagen IV; FN, fibronectin; LN, laminin.

To define the properties of the EC matrix, the topography of collagen I‐coated substrate and the EC matrix was compared using SEM Results revealed that the surfaces of collagen I‐coated substrate is covered with many of granules and fibre fines. (Fig. 1A). On the other hand, the suprastructure of the EC matrix was composed of fibrillar network which built on a layer of dense substance. The fibres on the EC matrix were thicker, which aligned with parallel arrangement or branch patterns within the matrix network (Fig. 1B). Immunofluorescence staining confirmed the major ECM components of the EC matrix, including FN, collagen I and collagen IV. FN fibres aligned with parallel or branch pattern, collagen I appeared dense dots, and collagen IV presented with net structures (Fig. 1C). Considered the ECM protein in FBS or the HUVECs lysis soon after the cells were lysed could be efficiently attached on the coverslips. The surface views of the gelatin‐treated coverslips cultured with endothelial cell growth medium containing 10% FBS or cell lysis were examined using SEM. Results showed that the ECM protein in FBS (Fig. S2A) or cell lysis (Fig. S2B) could not form the fibrillar suprastructure on the surface of the gelatin‐treated coverslips. The topography of the EC matrix did not lose after cultured with hepatocyte maintenance medium containing 10% FBS (Fig. S2C). Together, these data indicated that the ECM secreted by HUEVCs was stably anchored and formed a fibrillar suprastructure with an abundance of FN, collagen I and collagen IV.

### EC matrix promotes the metabolic functional maturation of hASC‐HLCs

To assess the ability of EC matrix to influence functional maturation of hASC‐HLCs, the cells were seeded onto the EC matrix or the collagen I‐coated substrate for different time‐points. Cell morphology under phase‐contrast microscopy showed that hASC‐HLCs exhibited more identical stereoscopic with a polygonal shape on the EC matrix than the collagen I‐coated substrate after they were cultured for 72 hrs (Fig. S3A).

**Figure 2:**
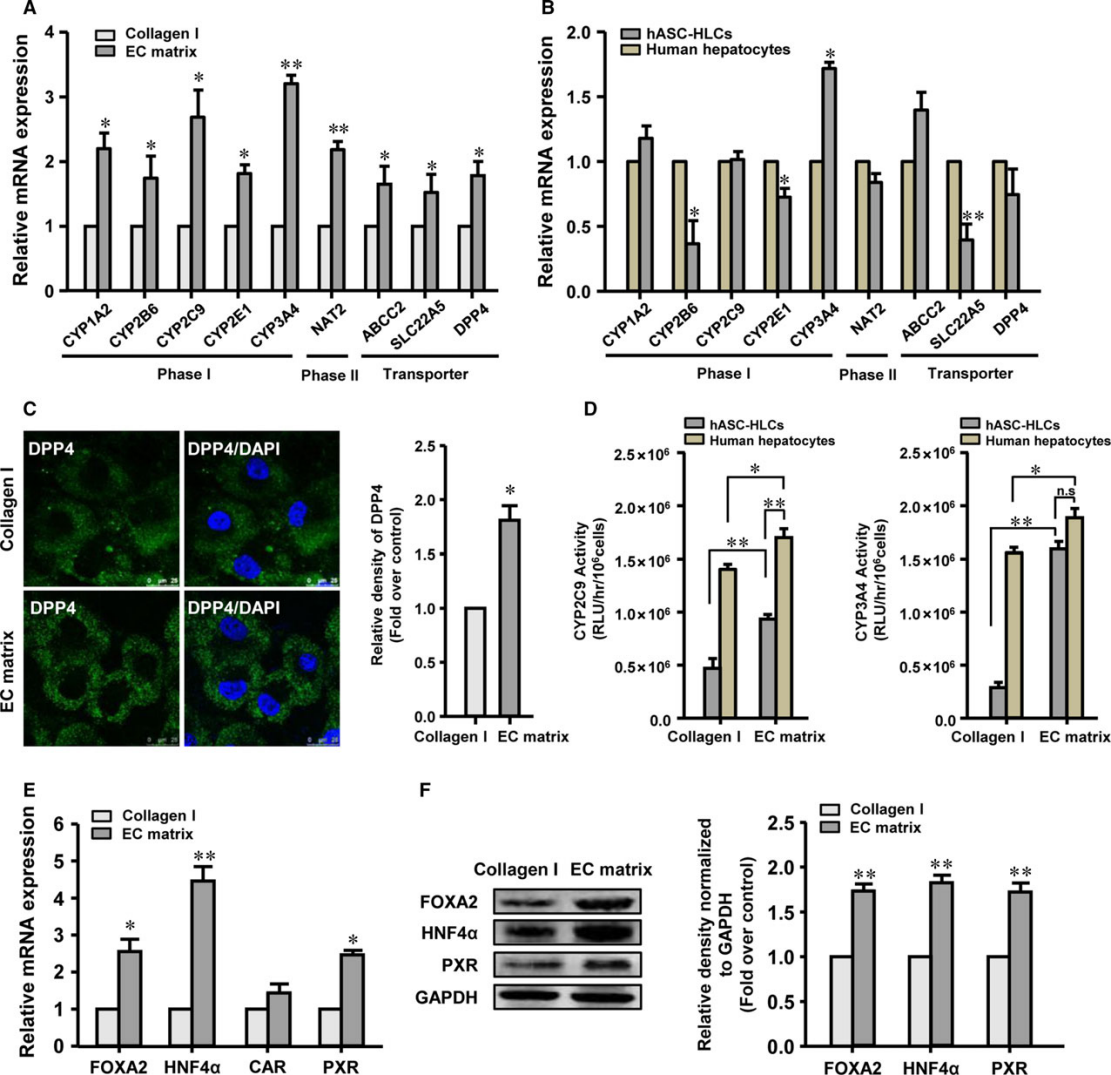
EC matrix promotes the metabolic functional maturation of hASC‐HLCs. Real‐time RT‐PCR analyses the expression of phase I drug‐metabolizing enzymes, phase II drug‐metabolizing enzymes and drug transporters in hASC‐HLCs cultured on the EC matrix and the collagen I‐coated substrate (**A**), or in hASC‐HLCs or human hepatocytes cultured on the EC matrix (**B**). Results are given as a mean ± S.D of three independent experiments with three batches of human hepatocytes and hASC‐HLCs from four different donors separately. Statistical significance of hASC‐HLCs cultured on the EC matrix compared to the collagen I‐coated substrate (**A**) or human hepatocytes cultured on the EC matrix, *P < 0.05; **P < 0.01. (**C**) Immunofluorescence staining and relative fluorescence density analysis of human DPP4 protein levels in hASC‐HLCs. Scale bars, 25 μm. Statistical significance of hASC‐HLCs cultured on the EC matrix compared to the collagen I‐coated substrate, *P < 0.05. (**D**) The activities of CYP2C9 and CYP3A4 after induced by rifampicin were assessed in hASC‐HLCs and human hepatocyte cultured on the EC matrix and the collagen I‐coated substrate. *P < 0.05; **P < 0.01; n.s., not significantly different. (**E**) The mRNA levels of FOXA2, HNF4α, CAR and PXR in hASC‐HLCs were measured using real‐time RT‐PCR. Statistical significance compared to the collagen I‐coated substrate, *P < 0.05, **P < 0.01. (**F**) The protein levels of FOXA2, HNF4α and PXR were examined by Western blot. Statistical significance compared to the collagen I‐coated substrate, *P < 0.05, **P < 0.01. Collagen I, collagen I‐coated substrate; RLU, relative luminescent unit.

Then, the hepatic‐specific functional and metabolic gene expression patterns in hASC‐HLCs were determined after they were cultured on different substrates for 72 hrs. Results showed that the mRNA levels of albumin (ALB), phosphoenolpyruvate carboxykinase 2 (PCK2) and carbamoyl‐phosphate synthase 1 (CPS1) in the hASC‐HLCs cultured on the EC matrix were not significantly different from the cells cultured on the collagen I‐coated substrate (Fig. S3B). However, the expression levels of phase I drug‐metabolizing enzymes—CYP1A2, CYP2B6, CYP2C9, CYP2E1 and CYP3A4—phase II drug‐metabolizing enzymes—N‐acetyltransferase 2 (NAT2)—and drug transporters—ATP‐binding cassette, subfamily C (CFTR/MRP), member 2 (ABCC2), solute carrier family 22 (organic cation/carnitine transporter), member 5 (SLC22A5) and dipeptidyl peptidase 4 (DPP4)—in hASC‐HLCs cultured on the EC matrix were significantly higher than in the cells cultured on the collagen I‐coated substrate (Fig. 2A). In addition, the EC matrix also promotes the up‐regulation of the metabolic gene expression in the human hepatocytes (Fig. S3C). The expression levels of CYP1A2, CYP2C9, CYP3A4, NAT2, ABCC2 and DPP4 in hASC‐HLCs cultured on the EC matrix were similar to the human hepatocytes cultured on the EC matrix (Fig. 2B). However, the mRNA levels of CYP2B6, CYP2E1 and SLC22A5 in hASC‐HLCs cultured on the EC matrix were still lower than the mRNA levels in the human hepatocytes cultured on the EC matrix (Fig. 2B). Thereafter, the key metabolic gene *CYP2C9* and *CYP3A4* expressions in adult hepatocytes were evaluated in hASC‐HLCs after they were cultured on the EC matrix for different time‐points. The results indicated that the EC matrix induced a peak expression of these markers on day 3 (Fig. S3D). Then, the metabolic properties of the hASC‐HLCs cultured on the EC matrix were analysed further at day 3.

Immunofluorescence staining confirmed that the protein level of DPP4, the specific transporter localized on the apical membrane of hepatocyte, in the hASC‐HLCs cultured on the EC matrix was significantly higher than that in the cells cultured on the collagen I‐coated substrate (Fig. 2C).

To further evaluate the function of the drug metabolism activities of the hASC‐HLCs cultured on the EC matrix, we focused on CYP2C9 and CYP3A4. Result showed that the basal metabolic activities of CYP2C9 and CYP3A4 enzymes in the hASC‐HLCs cultured on the EC matrix were not significantly different from the cells cultured on the collagen I‐coated substrate (Fig. S3E). However, the metabolic activities of CYP2C9 and CYP3A4 enzymes after induction by rifampicin in the hASC‐HLCs cultured on the EC matrix were 2.3‐ and 4.1‐fold higher than in the cells cultured on the collagen I‐coated substrate (Fig. 2D). The induction folds of the metabolic activities of CYP2C9 and CYP3A4 in hASC‐HLCs cultured on the EC matrix were 40‐ and 106‐fold separately. More importantly, the metabolic activities of CYP3A4 in the hASC‐HLCs cultured on the EC matrix were equivalent to those of CYP3A4 in the human hepatocytes cultured on EC matrix (Fig. 2D).

Previous studies have highlighted that hepatic metabolism is controlled by the transcription factor FOXA2 34 and by a set of nuclear receptors, including HNF4α, PXR and CAR 12, 35. Therefore, we suggested that these factors may be modulated by the EC matrix. The results showed that the mRNA expression of FOXA2, HNF4α and PXR in the hASC‐HLCs cultured on the EC matrix had an expected 2.6‐, 4.5‐ and 2.56‐fold increase over the cells cultured on the collagen I‐coated substrate. The expression of another nuclear receptor, CAR, in the hASC‐HLCs cultured on the EC matrix was not different from the cells cultured on the collagen I‐coated substrate (Fig. 2E). Similarly, the protein levels of FOXA2, HNF4α and PXR in the hASC‐HLCs cultured on the EC matrix were significantly higher than those in the cells cultured on the collagen I‐coated substrate (Fig. 2F). These findings suggest that the EC matrix modulated the expression of FOXA2, HNF4α and PXR and consequently modified the expression of several of its downstream functional targets, promoting the metabolic functional maturation of hASC‐HLCs.

### Selective silencing of FN attenuated the effect of the EC matrix

To investigate the potential components in the EC matrix contributing to the functional maturation of hASC‐HLCs, the FN was selectively silenced by delivering FN‐specific siRNA to HUVECs. The results showed that FN expression was successfully reduced by 80% compared to the control siRNA group at the same mRNA levels (Fig. S4A). Immunofluorescence staining and quantitative analyses showed that the protein level of FN expression in HUVECs in the FN siRNA group was significantly lower than that of the cells in the control siRNA group (Fig. S4B). SEM revealed that the fibrillar suprastructure was disrupted in the EC matrix from the FN‐deficient HUVECs (Fig. S4C).

**Figure 3:**
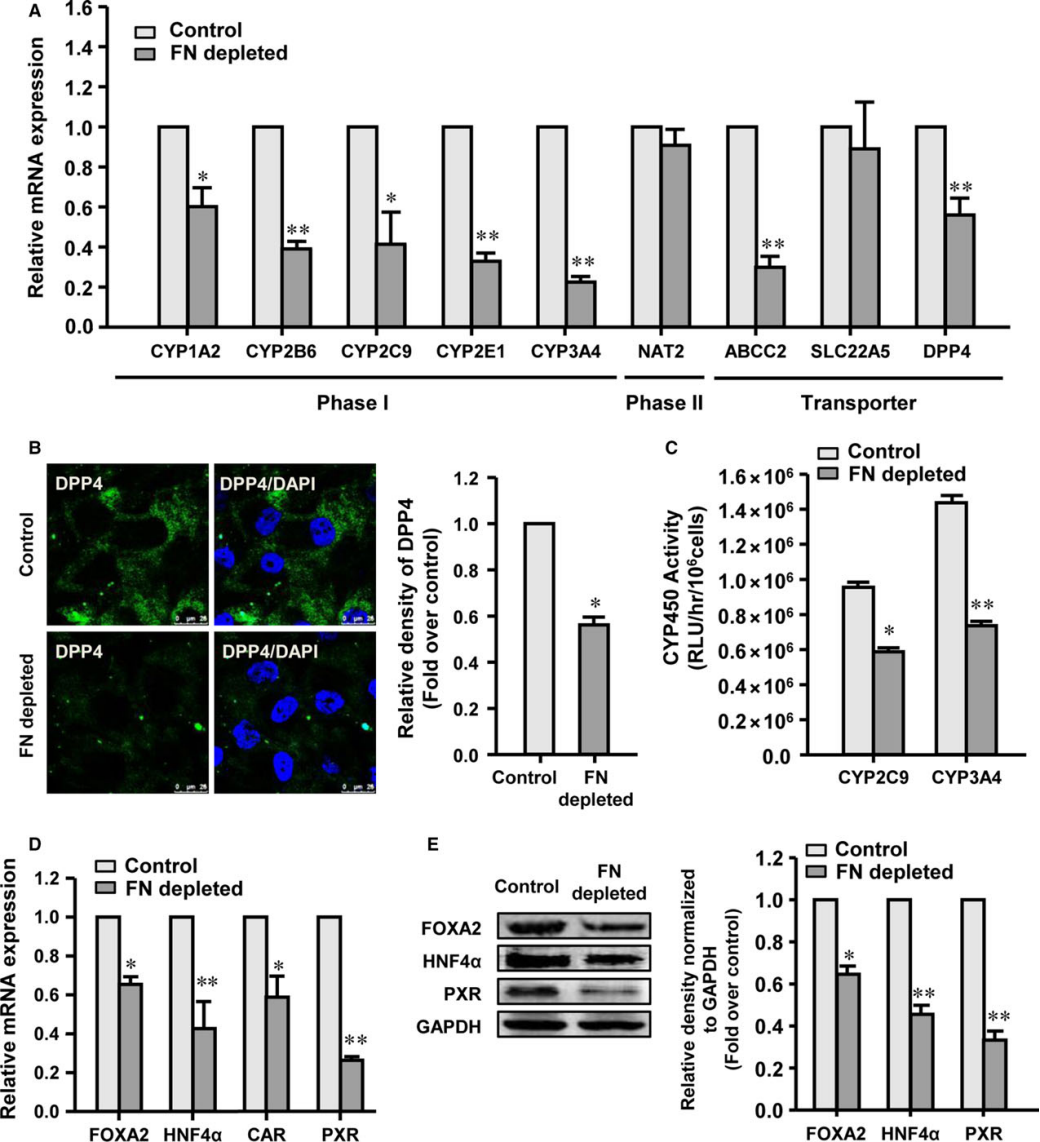
Depletion of FN in EC matrix attenuates the effect on hepatic maturation. (**A**) Real‐time RT‐PCR analyses the expression of phase I and phase II drug‐metabolizing enzymes and drug transporters in hASC‐HLCs cultured on the control EC matrix and the FN‐depleted EC matrix for 72 hrs. (**B**) Immunofluorescence staining and relative fluorescence density analyses of human DPP4 in hASC‐HLCs. Scale bars, 25 μm. (**C**) The activities of CYP2C9 and CYP3A4 after induced by rifampicin were assessed in hASC‐HLCs. (**D**) The mRNA levels of FOXA2, HNF4α, CAR and PXR in hASC‐HLCs were measured using real‐time RT‐PCR. (**E**) The protein levels of FOXA2, HNF4α and PXR were examined by Western blot. Statistical significance compared to the control siRNA, *P < 0.05; **P < 0.01. Control, the control EC matrix; FN, fibronectin; FN depleted, the FN‐depleted EC matrix; RLU, relative luminescent unit.

By reducing the FN expression level using siRNA, the effects of the FN‐depleted EC matrix derived from the FN siRNA‐treated HUVEC, the control EC matrix derived from the control siRNA‐treated HUVEC and the untreated EC matrix derived from the untreated HUVEC on the functional maturation of hASC‐HLCs were assessed. The results showed that the mRNA levels of the metabolic genes in hASC‐HLCs cultured on the control EC matrix were not significantly different than the cells cultured on the untreated EC matrix (Fig. S4D). However, the FN deficient in EC matrix had significantly decreased expression levels of phase I drug‐metabolizing enzymes (CYP1A2, CYP2B6, CYP2C9, CYP2E1 and CYP3A4) and drug transporters (ABCC2 and DPP4) in hASC‐HLCs, but the expression levels of phase II drug‐metabolizing enzymes (NAT2) and drug transporters (SLC22A5) were not affected (Fig. 3A). Immunofluorescence staining confirmed that the protein level of DPP4 in the hASC‐HLCs cultured on the FN‐depleted EC matrix was significantly lower than in the cells cultured on the control EC matrix (Fig. 3B). The metabolic activities of CYP2C9 and CYP3A4 upon rifampicin induction in the hASC‐HLCs cultured on the FN‐depleted EC matrix were also found to be significantly decreased than in the cells cultured on the control EC matrix (Fig. 3C). The basal CYP450 activities in the hASC‐HLCs cultured on the FN‐depleted EC matrix showed a similar extent to the cells cultured on the control EC matrix (Fig. S4E). These results suggested that FN in the EC matrix plays a key role in promoting the functional maturation of hASC‐HLCs.

To confirm our findings, the CYP450 activities in the hASC‐HLCs after induced by rifampicin were analysed when the cells were cultured on FN‐coated substrate and collagen I plus FN‐coated substrate. The results showed that the metabolic activities of CYP2C9 and CYP3A4 in the hASC‐HLCs cultured on the FN‐coated substrate, and collagen I plus FN‐coated substrate, were also significantly lower than in the cells cultured on the EC matrix (Fig. S4F). SEM showed that FN molecules could not assemble into fibrils when they were coated on the gelatin‐treated substrate (Fig. S2D). These results indicated that the metabolic maturation of hASC‐HLCs cultured on the EC matrix depends on the specific fibrillar FN in the cell‐derived matrix.

To explore the extent of characteristic expression of hepatic transcription factor and nuclear receptors, the mRNA levels of FOXA2, HNF4α, PXR and CAR were evaluated. The results showed that the mRNA levels of FOXA2, HNF4α and PXR in the hASC‐HLCs cultured on the FN‐depleted EC matrix showed expected 37%, 59% and 73% decreases compared to the cells cultured on the control EC matrix. Moreover, the expression of CAR was also found to be decreased in the hASC‐HLCs cultured on the FN‐depleted EC matrix compared to that observed in the cells cultured on the control EC matrix (Fig. 3D). The protein levels of FOXA2, HNF4α and PXR in the hASC‐HLCs cultured on the FN‐depleted EC matrix were significantly lower than those in the cells cultured on the control EC matrix (Fig. 3E). Taken together, these results indicated that the fibrillar FN in the EC matrix plays a role in promoting the functional maturation of hASC‐HLCs.

### Integrin signalling activation modulates the effects of the EC matrix on hASC‐HLCs

**Figure 4:**
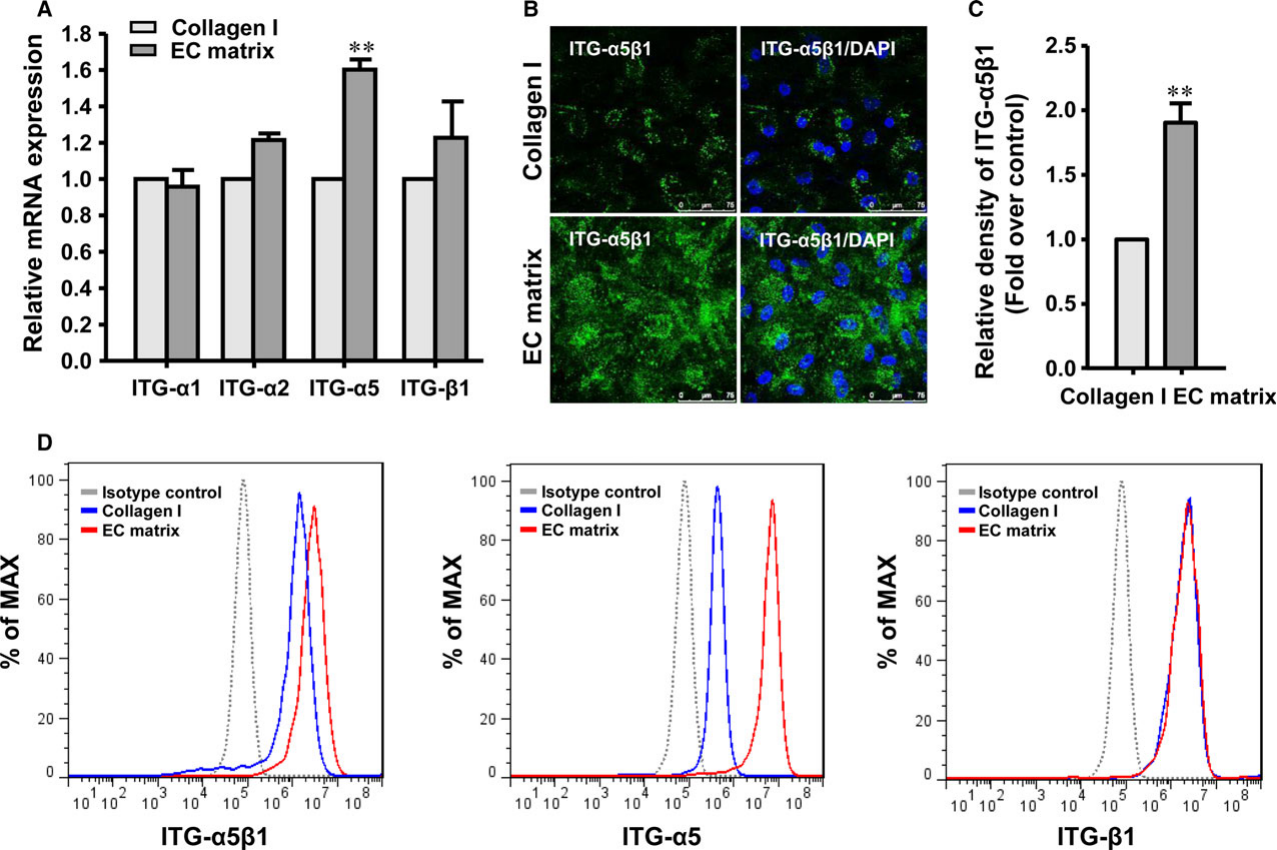
Expression of integrin in hASC‐HLCs. (**A**) Real‐time RT‐PCR analysis of the subunits α1, α2, α5 and β1 integrin mRNA levels in hASC‐HLCs cultured on the EC matrix and the collagen I‐coated substrate. (**B**) Immunofluorescence staining for α5β1 integrin in hASC‐HLCs was examined. Scale bars, 75 μm. (**C**) Quantitative analysis of α5β1 integrin was determined using Image J. (**D**) Flow cytometric analysis of subunits α5, β1 integrin and α5β1 integrin expression in hASC‐HLCs. Statistical significance compared to the collagen I, **P < 0.01. Collagen I, collagen I‐coated substrate, ITG, integrin.

In an attempt to understand the mechanism by which the EC matrix promotes the functional maturation of hASC‐HLCs, we next examined the expression of integrins, the receptors of the ECM in cells. The mRNA levels of the subunits α1, α2, α5 and β1 integrin, which are mainly expressed in hepatocytes 31, 36, were determined in hASC‐HLCs after the cells were plated on the EC matrix or the collagen I‐coated substrate for 72 hrs. As shown in Figure 4A, the expression of the α5 integrin was significantly increased in the hASC‐HLCs cultured on the EC matrix. Immunofluorescence staining and quantitative analyses showed that the protein level of activated α5β1 integrin in hASC‐HLCs cultured on the EC matrix was also significantly higher than that in the cells cultured on the collagen I‐coated substrate (Fig. 4B and 4C). The expression of α5, β1 and α5β1 integrin in the hASC‐HLCs cultured on the different substrates was further examined by flow cytometric analysis. Increases in the percentages of the α5 integrin in the hASC‐HLCs from 77.2% (cultured on the collagen I‐coated substrate) to 97.5% (cultured on the EC matrix) and of the α5β1 integrin from 83.7% (cultured on collagen I‐coated substrate) to 93.6% (cultured on the EC matrix) were observed, whereas the levels of subunit β1 integrin present in the hASC‐HLCs did not vary between the two groups (Fig. 4D).

To investigate the potential role of the α5β1 integrin in the EC matrix on the functional maturation of hASC‐HLCs, the α5 integrin was silenced by delivering siRNA to the hASC‐HLCs. The results showed that the mRNA level of the α5 integrin was successfully reduced by 50% compared to the control siRNA group (Fig. S5A). Immunofluorescence staining and quantitative analyses showed that the protein level of the activated α5β1 integrin in the α5 integrin siRNA group was significantly lower than that of the cells in the control siRNA group (Fig. S5B).

**Figure 5:**
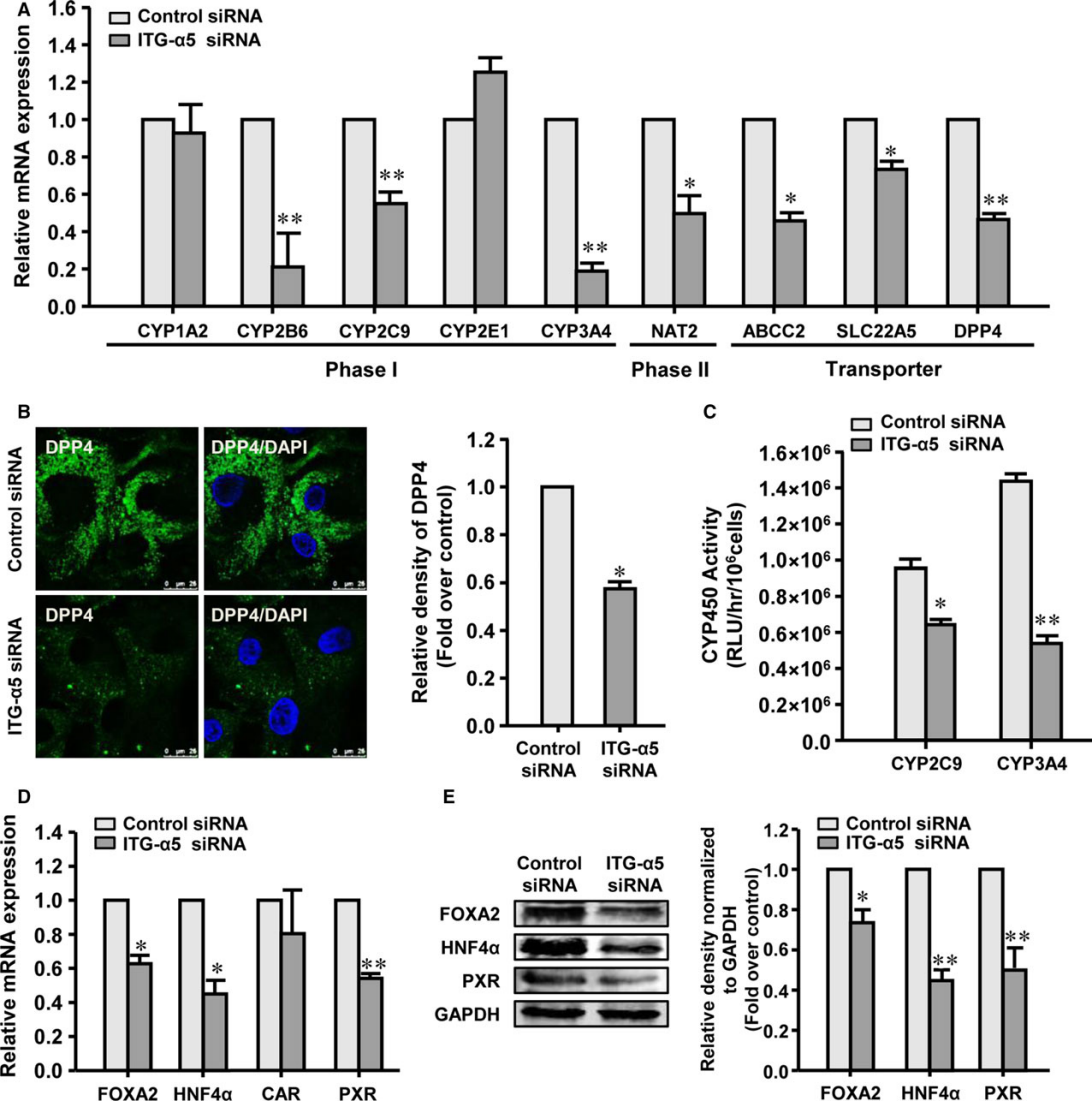
Depletion of α5 integrin in hASC‐HLCs attenuates the effect of the EC matrix on hepatic maturation. (**A**) Real‐time RT‐PCR analyses the expression of phase I and phase II drug‐metabolizing enzymes and drug transporters in hASC‐HLCs‐α5 integrin siRNA and hASC‐HLCs‐control siRNA cultured on the EC matrix for 72 hrs. (**B**) Immunofluorescence staining of human DPP4 in hASC‐HLCs. Relative fluorescence densities of proteins were quantitatively assayed using Image J. Scale bars, 25 μm. (**C**) The activities of CYP2C9 and CYP3A4 after induced by rifampicin were assessed in hASC‐HLCs‐α5 integrin siRNA and hASC‐HLCs‐control siRNA. (**D**) The mRNA levels of FOXA2, HNF4α, CAR and PXR were measured using real‐time RT‐PCR. (**E**) The protein levels of FOXA2, HNF4α and PXR were examined by Western blot. Statistical significance compared to the control siRNA, *P < 0.05; **P < 0.01. ITG, integrin; RLU, relative luminescent unit.

The expression and activity of the CYP450 enzymes in the hASC‐HLCs were assessed in the siRNA‐treated cells with reduced α5 integrin expression levels. The results showed that the hASC‐HLCs deficient in α5 integrin expression had significantly decreased mRNA levels of phase I drug‐metabolizing enzymes (CYP2B6, CYP2C9 and CYP3A4), phase II drug‐metabolizing enzyme (NAT2) and drug transporters (ABCC2, SLC22A5, and DPP4), but the expression levels of phase I drug metabolism enzymes (CYP1A2 and CYP2E1) were not affected (Fig. 5A).

Immunofluorescence staining confirmed that the protein level of DPP4 in the hASC‐HLCs‐integrin α5 siRNA was significantly lower than that in the hASC‐HLCs‐control siRNA (Fig. 5B). The metabolic activities of CYP2C9 and CYP3A4 in the hASC‐HLCs‐integrin α5 siRNA were decreased by 34% and 60%, respectively, compared to those in the hASC‐HLCs‐control siRNA (Fig. 5C). Likewise, the basal metabolic activities of CYP2C9 and CYP3A4 in the hASC‐HLCs‐integrin α5 siRNA were not significantly different compared to those in the hASC‐HLCs‐control siRNA (Fig. S5C).

The mRNA levels of FOXA2, HNF4α and PXR in the hASC‐HLCs‐integrin α5 siRNA significantly decreased compared to the hASC‐HLCs‐control siRNA, but the expression of CAR was not affected (Fig. 5D). The protein levels of FOXA2, HNF4α, and PXR in the hASC‐HLCs‐integrin α5 siRNA significantly decreased compared to those in the hASC‐HLCs‐control siRNA (Fig. 5E). These results suggest that the effects of the EC matrix on the metabolic functional maturation of hASC‐HLCs may occur through an α5β1 integrin‐mediated signalling pathway.

### EC matrix promotes the functional maturation of hASC‐HLCs *via* Src phosphorylation

**Figure 6:**
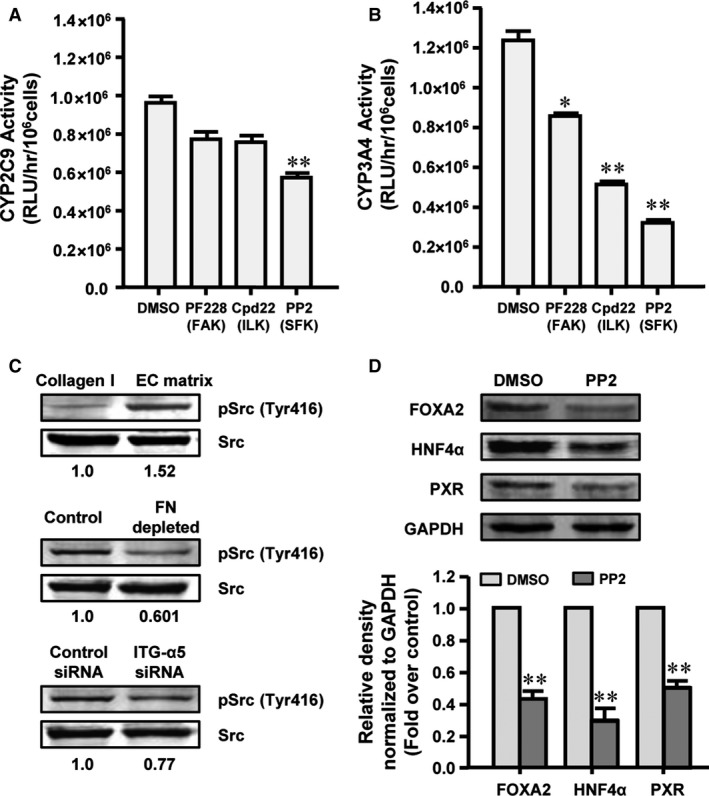
EC matrix promotes hepatic metabolic functional maturation dependent on Src phosphorylation. The activities of CYP2C9 (**A**) and CYP3A4 (**B**) after induced by rifampicin were assessed in hASC‐HLCs cultured on the EC matrix, which were treated with FAK, SFK and ILK inhibitors for 15 hrs. (**C**) The protein levels of p‐Src (Tyr416) and total Src were measured by Western blot in hASC‐HLCs cultured on the EC matrix and the collagen I‐coated substrate for 72 hrs, or cultured on the FN‐depleted and control EC matrix, or in α5 integrin siRNA‐treated and control siRNA‐treated hASC‐HLCs, which were cultured on the EC matrix. (**D**) The protein levels of FOXA2, HNF4α and PXR in hASC‐HLCs were examined by Western blot. Statistical significance compared to the control, *P < 0.05; **P < 0.01. Collagen I, collagen I‐coated substrate; control, the control EC matrix; FAK, focal adhesion kinase; FN depleted, the FN‐depleted EC matrix; ILK, integrin‐linked kinase; ITG, integrin; RLU, relative luminescent unit; SFK, Src family kinase.

To determine the functional downstream signalling molecules, the metabolic activities of CYP2C9 and CYP3A4 after induced by rifampicin in the hASC‐HLCs cultured on the EC matrix were assessed in the presence or absence of ILK, FAK and SFK inhibitors for 15 hrs. The results showed that the activities of CYP2C9 and CYP3A4 were ~40% and ~75% attenuated by the pharmacological inhibition of SFK with PP2 (5 μM) (Fig. 6A and B). The activity of CYP3A4 was ~30% and ~58% attenuated by pharmacological inhibition of FAK with PF228 (1 μM) and ILK with Cpd22 (1 μM) (Fig. 6B). However, neither PF228 nor Cpd22 treatment prevented the activities of CYP2C9 (Fig. 6A). These results indicate that the effect of the EC matrix in regulating the functional maturation of hASC‐HLCs mainly depends on SFK, the core kinase of the Src pathway.

To confirm that the EC matrix activates SFK, the protein level of Src phosphorylated at tyrosine 416 (Tyr416) in the hASC‐HLCs cultured on the EC matrix or the collagen I‐coated substrate was evaluated. As expected, culturing cells on the EC matrix for 72 hrs increased the protein level of phospho‐Src (Tyr416) in the hASC‐HLCs by 1.52‐fold (Fig. 6C). Similarly, the EC matrix‐induced Src phosphorylation could be reduced by knocking down FN in the EC matrix and by knocking down α5 integrin in the hASC‐HLCs (Fig. 6C). Treatment with the SFK inhibitor PP2 significantly decreased the expression of FOXA2, HNF4α and PXR in hASC‐HLCs (Fig. 6D).

**Figure 7:**
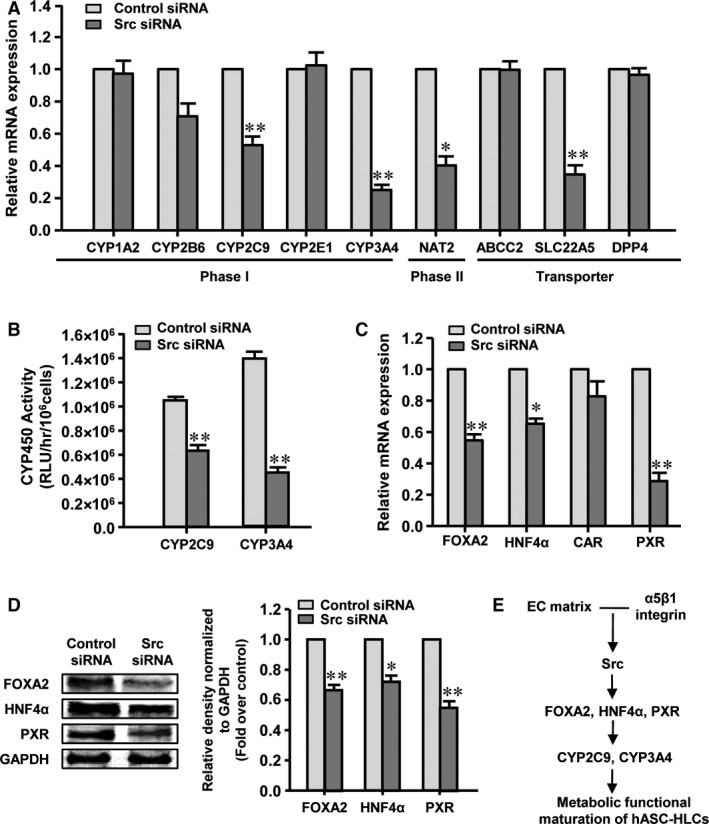
EC matrix promotes metabolic functional maturation of hASC‐HLCs dependent on Src signalling. (**A**) Real‐time RT‐PCR analyses the expression of phase I and phase II drug‐metabolizing enzymes and drug transporters in hASC‐HLCs‐Src siRNA and hASC‐HLCs‐control siRNA cultured on the EC matrix for 72 hrs. (**B**) The activities of CYP2C9 and CYP3A4 after induced by rifampicin were assessed in hASC‐HLCs‐Src siRNA and hASC‐HLCs‐control siRNA. (**C**) Real‐time RT‐PCR analyses the expression of FOXA2, HNF4α, CAR and PXR in hASC‐HLCs‐Src siRNA and hASC‐HLCs‐control siRNA. (**D**) The protein levels of FOXA2, HNF4α and PXR were examined by Western blot. (**E**) Schematic view of the EC matrix regulation of the metabolic functional maturation of hepatocyte. The interaction between the EC matrix and α5β1 integrin in hASC‐HLCs activates the Src signalling pathway and then mediates the up‐regulation expression of the FOXA2, HNF4α and PXR, which regulates the expression of the key metabolic functional genes, including CYP2C9, CYP3A4, and then promotes the metabolic functional maturation of hASC‐HLCs. Statistical significance compared to the control siRNA, *P < 0.05; **P < 0.01. Collagen I, collagen I‐coated substrate; ITG, integrin; RLU, relative luminescent unit.

To investigate the potential role of Src in the EC matrix on the functional maturation of hASC‐HLCs, the Src was silenced by delivering siRNA to the hASC‐HLCs. The results showed that the mRNA level and the protein level of the Src in Src siRNA‐treated hASC‐HLCs were found to be significantly reduced compared to the control siRNA group (Fig. S6A and B). The Src deficient in hASC‐HLCs significantly decreased the mRNA levels of phase I drug‐metabolizing enzymes (CYP2C9, and CYP3A4), phase II drug‐metabolizing enzyme (NAT2) and drug transporters (SLC22A5), but the expression levels of phase I drug‐metabolizing enzymes (CYP1A2, CYP2B6 and CYP2E1), and drug transporters (ABCC2 and DPP4) were not affected (Fig. 7A). The metabolic activities of CYP2C9 and CYP3A4 in the hASC‐HLCs‐Src siRNA were significantly lower than those in the hASC‐HLCs‐control siRNA (Fig. 7B). The mRNA levels and protein levels of FOXA2, HNF4α and PXR in the hASC‐HLCs‐Src siRNA significantly decreased compared to the hASC‐HLCs‐control siRNA (Fig. 7C and 7D). These results suggest that the effects of the EC matrix on the metabolic functional maturation of hASC‐HLCs depend on Src‐mediated signalling pathway.

Collectively, these results indicate that the EC matrix promotes the expression of α5 integrin in hASC‐HLCs; α5β1 integrin specifically interacts with FN in the EC matrix, activates the Src phosphorylation in the cells and then up‐regulates the expression of FOXA2, HNF4α and PXR, which promote the expression and activities of CYP2C9 and CYP3A4 that result in the metabolic functional maturation of hASC‐HLCs (Fig. 7E).

## Discussion

In this study, we identified the architectural properties and biochemical components of decellularized human HUVEC‐derived matrix. We provided evidence that the EC matrix modulated the expression of FOXA2, HNF4α and PXR and thus regulated the expression of several of their downstream functional targets, including CYP450 enzymes and transporters, promoting the metabolic functional maturation of hASC‐HLCs. We identified the relationship between the fibrillar FN in EC matrix and α5β1 integrin that mediated the metabolic functional maturation of hASC‐HLCs. We demonstrated that the EC matrix modulates hepatocyte metabolic functional maturation through Src phosphorylation. We proposed a molecular mechanism linking the EC matrix and the metabolic functional maturation of hepatocytes, thereby identifying a new role of integrin.

Previous studies have shown that endothelial cells produce specific matrix proteins important in liver health and disease 37. However, the effect of EC matrix on hepatocyte differentiation and maturation is not clear. Here, we identified that the EC matrix was a meshwork of fibrillar microstructure with intensive dots on the surface, and FN, collagen I and collagen IV were the abundant components in the EC matrix. The components of the EC matrix were similar to those of the ECM located in the space of Disse in normal liver. Conceptually, a cell‐made matrix has the advantage that processes such as post‐translational modification, fibrillogenesis and the aligned deposition and remodelling of matrix molecules are conducted in a physiologically relevant manner 18. Therefore, we suggest that the EC matrix might mimic the ECM produced *in vivo* by liver endothelial cells and have the potential to promote or maintain the differentiation and functional maturation of hepatocytes. Thus, our study aimed to decipher the effects and mechanisms of the EC matrix response in hepatocyte maturation.

First, we have shown that the EC matrix selectively promotes the expression levels of metabolizing enzymes including phase I metabolizing enzymes (CYP1A2, CYP2B6, CYP2C9, CYP2E1 and CYP3A4), a phase II metabolizing enzyme (NAT2) and transporters (ABCC2, SLC22A5 and DPP4) in hASC‐HLCs. These proteins have been extensively studied in hepatocytes because of their implication in drug metabolism and toxicologic processes in adult hepatocytes 38. More importantly, the activities of CYP2C9 and CYP3A4 in the hASC‐HLCs cultured on the EC matrix were found to be significantly increased compared to those in the cells cultured on the collagen I‐coated substrate. Previous studies have highlighted that hepatic metabolism is controlled by FOXA2 34 and a set of nuclear receptors, including hepatocyte HNF4α, PXR and CAR 12, 35. Therefore, we suggested that these nuclear receptors may be modulated by EC matrix. The results confirmed that the EC matrix modulated the expression of FOXA2, HNF4α and PXR, and thus the expression of several of its downstream functional targets, promoting the activities of CYP2C9 and CYP3A4.

Second, we have shown that selective silencing of FN attenuated the expression of metabolizing enzyme CYP450 and nuclear receptors, and it attenuated the activities of CYP2C9 and CYP3A4 in the hASC‐HLCs cultured on the EC matrix. Although FN has been studied as a large adhesive glycoprotein for more than two decades, the effect of this remarkably complex molecule in liver development and disease is still controversial 39, 40. Accumulating evidence proposes that FN not only plays important roles in cell adhesion and migration but is also critical for controlling the organization and composition of the ECM and mediates a wide variety of cellular interactions with the ECM that regulate cell differentiation 41, 42. However, the functional contribution of FN, particularly in the ECM, to hepatocyte maturation remains largely unknown. Our study indicated that FN is crucial in the role of the EC matrix to regulate the functional maturation of hASC‐HLCs.

Additionally, we have shown that silencing α5 integrin significantly decreased the expression of metabolizing enzyme CYP450 and nuclear receptors and decreased the activities of CYP2C9 and CYP3A4 in the hASC‐HLCs cultured on the EC matrix. Integrins, a protein family that comprises 18 α‐subunits and eight β‐subunits in mammals, play a pivotal role in mediating the interactions between different cell types and the ECM proteins 43. α5β1 integrin is a specific FN receptor, which is also the predominant integrin isoform in liver 36. Although α5β1 integrin has been confirmed mainly to be involved in focal adhesions, recent studies provided evidence that α5β1 integrin may be sensors for tauroursodeoxycholic acid in hepatocytes 44 or may respond to the migration of hepatic progenitor cells 45. Considering our data, α5β1 integrin is capable of eliciting a plethora of signalling responses that affect a host of functional outcome, which may be dependent on the different types of cells. Likewise, we noted the variations in the effects on hASC‐HLCs after treated with the siRNA. Knockdown of FN decreased all the phase I metabolizing enzymes; however, knockdown of the α5 integrin subunit decreased most of phase I metabolizing enzymes, but did not affect the expression of CYP1A2 and CYP2E1. Also, the reduced of FN decreased all four transcription factors, but the α5 siRNA treatment did not significantly affect the CAR. FN binds to not only integrins but also ECM molecules, such as collagen and heparan sulphate proteoglycans, and growth factors, such as HGF and VEGF 14. This suggests that the role of FN may not exclusively occur through the α5 integrin pathway.

Finally, the Src signalling pathway highlights the coupling between the EC matrix, FN and α5β1 integrin and hepatocyte maturation. Earlier studies have established that FAK, SFK and ILK regulated crosstalk between cells and the ECM 46, 47, 48. In this study, using their reported concentrations of the same inhibitors, FAK (1 μM PF228), SFK (5 μM, PP2) and ILK (1 μM Cpd22) 49, and the Src siRNA, we observed that inhibiting phosphorylation and the expression of Src dramatically mainly decreases the expression and activities of CYP3A4 and CYP2C9 and the protein levels of FOXA2, HNF4α and PXR.

Taken together, we demonstrated EC matrix as an important factor in the regulation of the metabolic functional maturation of hepatocyte. Although the molecular mechanism is still not well understood, this study showed that the α5β1 integrin‐activated Src phosphorylation signalling pathway mediated the interaction between extrinsic ECM signals and the expression of FOXA2, HNF4α and PXR in the hASC‐HLCs, thus resulting in up‐regulation activities of hepatic‐specific metabolic CYP450 enzymes.

## Conflict of Interest

The authors declare no conflict of interest.

## Author's contribution

X.G. and H.Z. designed the experiments, assembled data, analysed the data and wrote the manuscript. W.L. provided administrative support and performed the experiments. M.M. and X.L performed the experiments.

## Supporting information


**Figure S1** Expression of ECM components in HUVECs.

**Figure S2** The topography of different substrates.

**Figure S3** The properties of hASC‐HLCs and human hepatocytes on different substrates.

**Figure S4** Efficiency of depletion of FN in HUVECs and the effect on hepatic maturation.

**Figure S5** Efficiency of depletion of α5 integrin in hASC‐HLCs and the effect on hepatic metabolic maturation.

**Figure S6** Efficiency of depletion of Src in hASC‐HLCs.

**Table S1** List of antibodies used in the study.

**Table S2** Primers for Real‐Time RT‐PCR.

**Table S2** Primers for Real‐Time RT‐PCR.
