# Supplementary figures and images for: Endothelial cell‐derived matrix promotes the metabolic functional maturation of hepatocyte via integrin‐Src signalling
Source: J Cell Mol Med. 2017 May 4;21(11):2809–22. doi: 10.1111/jcmm.13195 (PMC5661128; doi:10.1111/jcmm.13195)

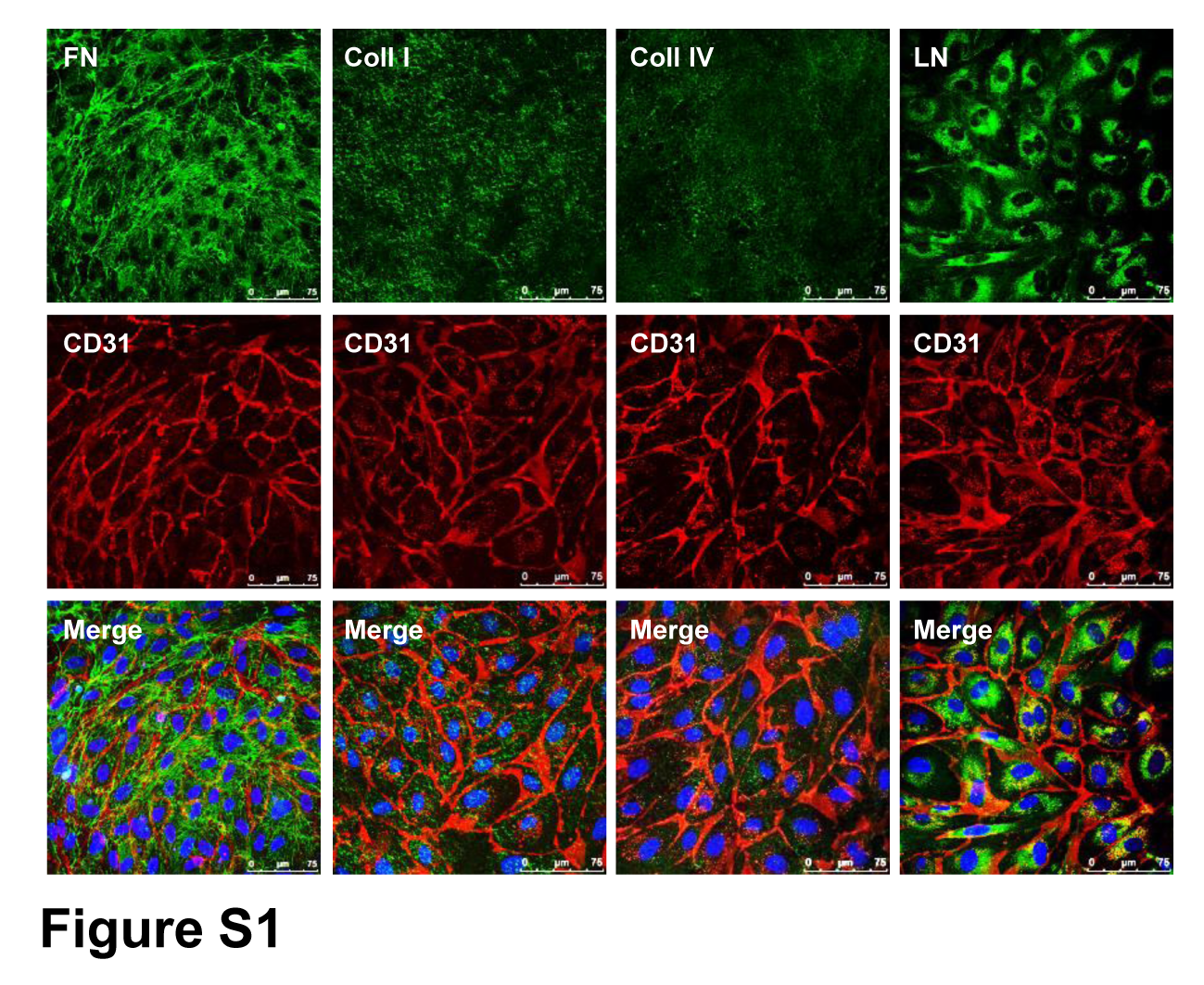

Supplement: Supplementary file 1 — Figure S1 Expression of ECM components in HUVECs. [file JCMM-21-2809-s001.tif]

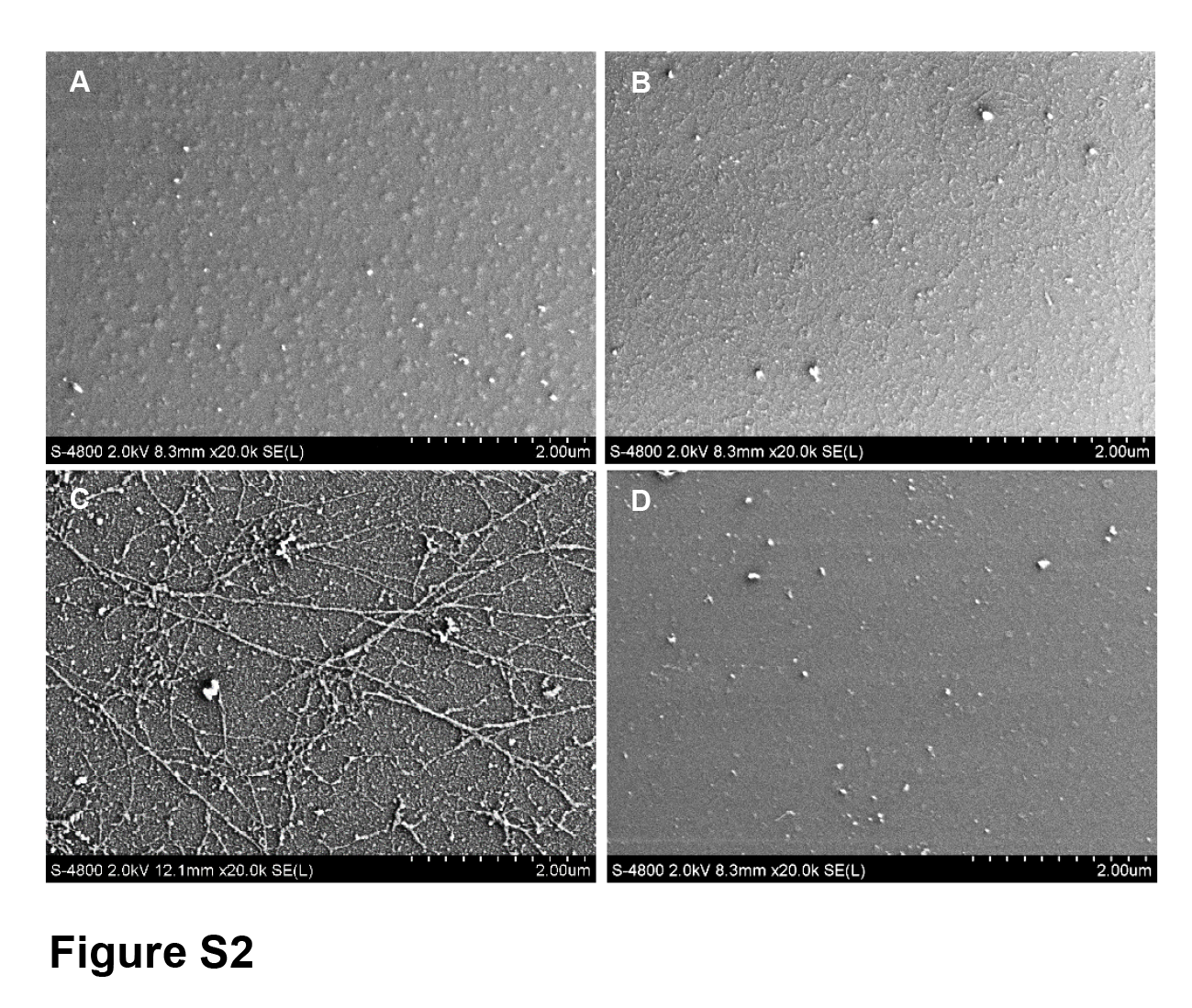

Supplement: Supplementary file 2 — Figure S2 The topography of different substrates. [file JCMM-21-2809-s002.tif]

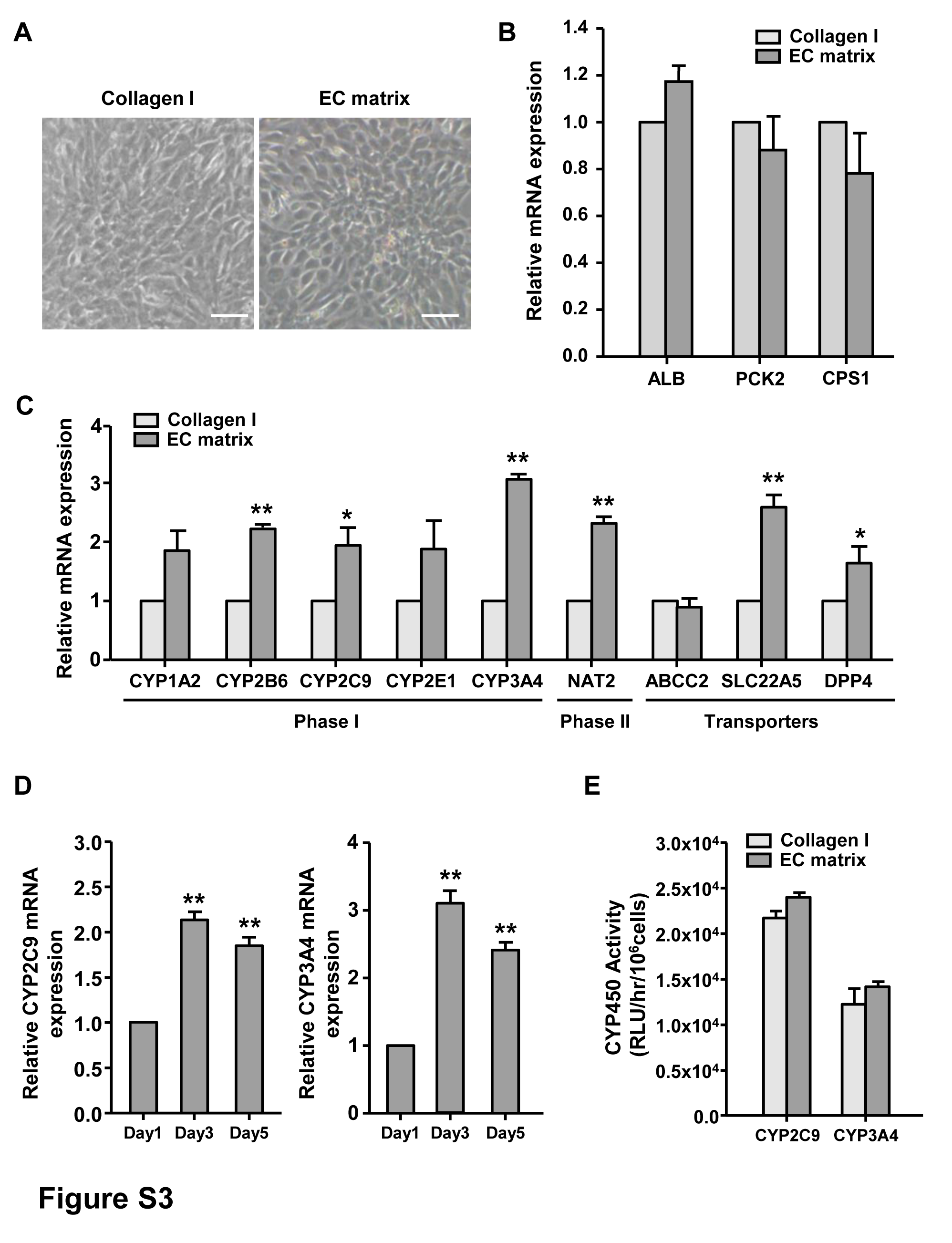

Supplement: Supplementary file 3 — Figure S3 The properties of hASC‐HLCs and human hepatocytes on different substrates. [file JCMM-21-2809-s003.tif]

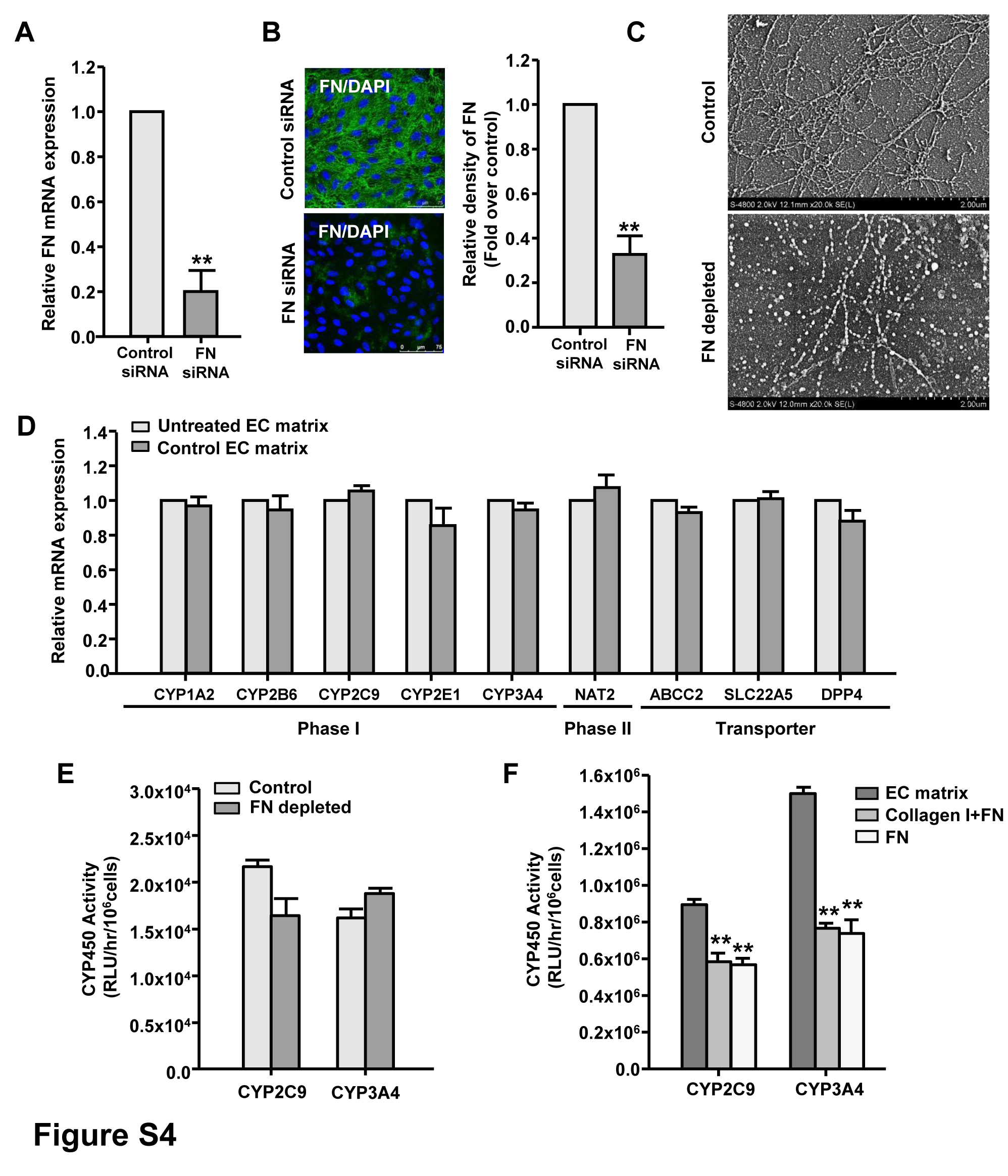

Supplement: Supplementary file 4 — Figure S4 Efficiency of depletion of FN in HUVECs and the effect on hepatic maturation. [file JCMM-21-2809-s004.tif]

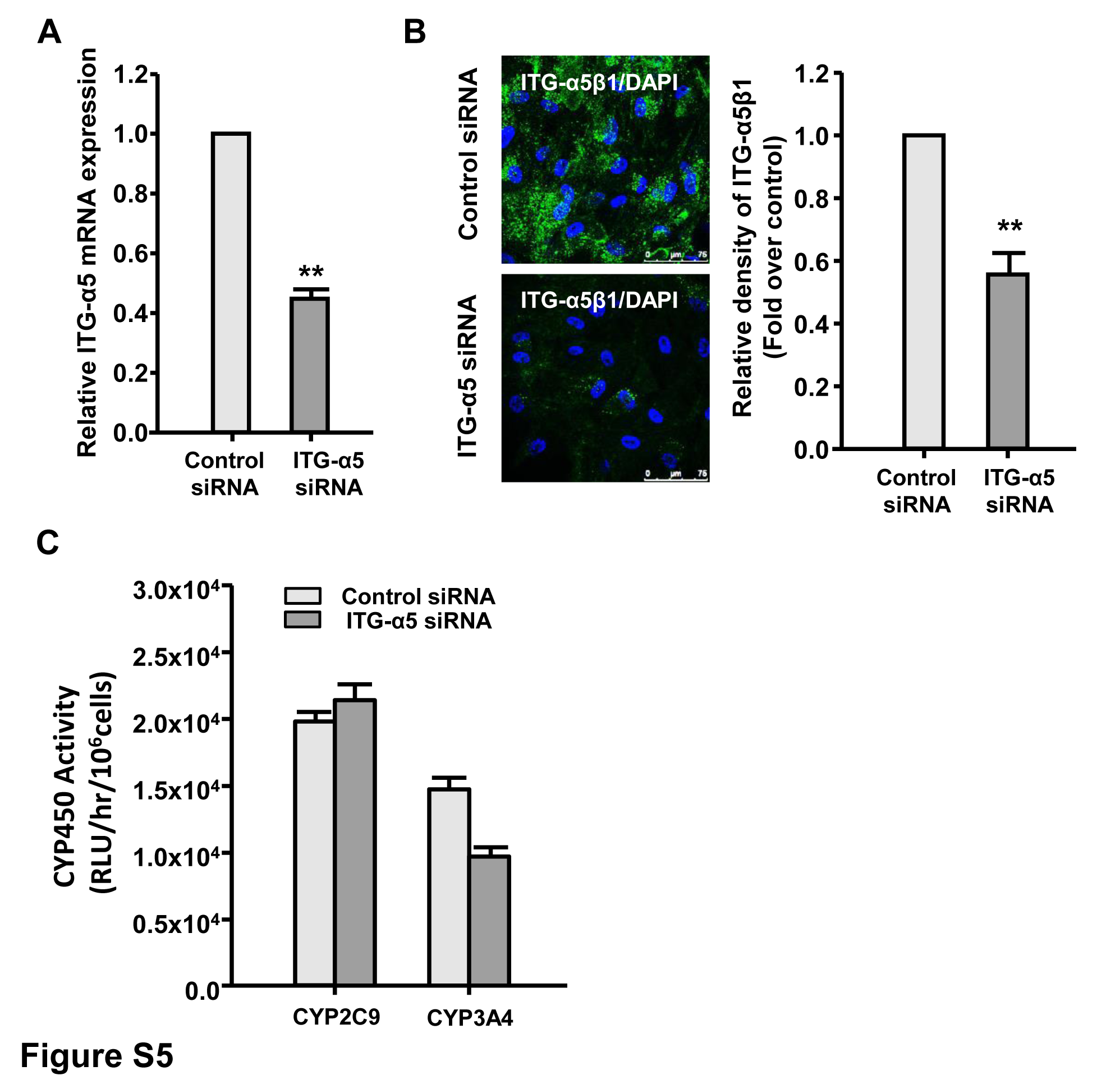

Supplement: Supplementary file 5 — Figure S5 Efficiency of depletion of α5 integrin in hASC‐HLCs and the effect on hepatic metabolic maturation. [file JCMM-21-2809-s005.tif]

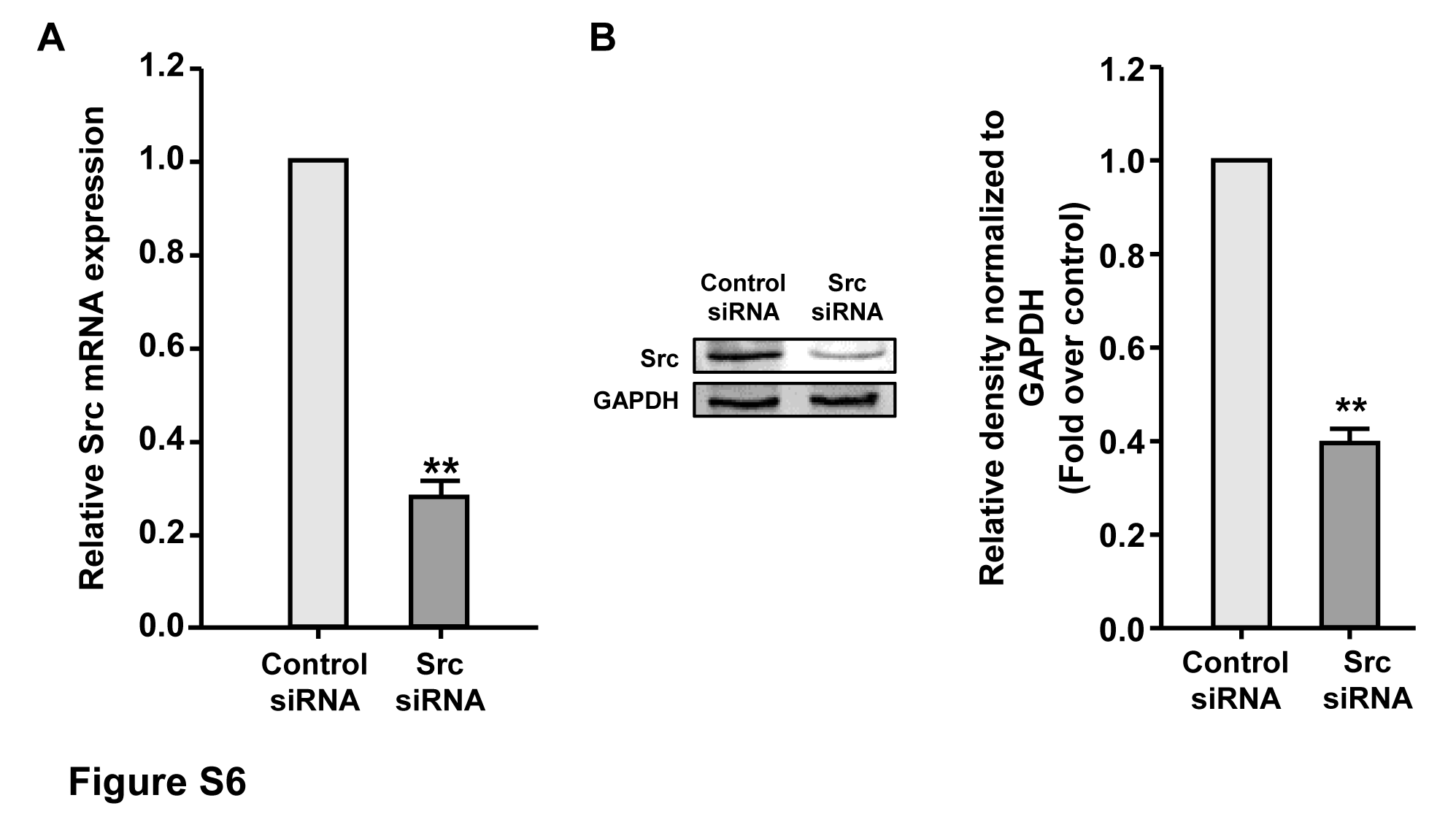

Supplement: Supplementary file 6 — Figure S6 Efficiency of depletion of Src in hASC‐HLCs. [file JCMM-21-2809-s006.tif]
