# Supplementary material for: Endothelial cell‐derived matrix promotes the metabolic functional maturation of hepatocyte via integrin‐Src signalling
Source: J Cell Mol Med. 2017 May 4;21(11):2809–22. doi: 10.1111/jcmm.13195 (PMC5661128; doi:10.1111/jcmm.13195)
Supplement: Supplementary file 7 — Table S1 List of antibodies used in the study. [file JCMM-21-2809-s007.doc]

Table S1. List of antibodies used in the study

Antibodies for Immunofluorescence

| **Primary Antibody** | **Dilutions** | **Company** | **Clonality** | **Cat. No.** |
| --- | --- | --- | --- | --- |
| Mouse anti-ITG α5β1 | 1:100 | Merck Millipore | monoclonal | MAB1999 |
| Mouse anti-CD31 | 1:3200 | Cell Signaling Technology | monoclonal | 3528 |
| Rabbit anti-CD31 | 1:100 | ZSGB-BIO | monoclonal | ZA-0568 |
| Mouse anti-DPP4 | 1:100 | Santa Cruz | monoclonal | SC-52469 |
| Rabbit anti-Collagen I | 1:100 | ZSGB-BIO | monoclonal | ZA-0616 |
| Mouse anti-Collagen IV | 1:100 | ZSGB-BIO | monoclonal | ZM-0081 |
| Mouse anti-fibronectin | 1:500 | Merck Millipore | monoclonal | MAB88916-C |
| Mouse anti-laminin | 1:100 | Santa Cruz | monoclonal | SC-74418 |

Antibodies for Western blot analysis

| **Primary Antibody** | **Dilution** | **Company** | **Clonality** | **Cat.No.** |
| --- | --- | --- | --- | --- |
| Rabbit anti-FOXA2 | 1:1000 | Abgent | Polyclonal(RB31713) | AP12031B |
| Rabbit anti-HNF4α | 1:1000 | Cell Signaling Technology | monoclonal | 3113 |
| Mouse anti-PXR | 1:200 | Santa Cruz | monoclonal | SC-48403 |
| Rabbit anti-Src | 1:1000 | Cell Signaling Technology | Polyclonal | 2109 |
| Rabbit anti-phospho-Src(Tyr416) | 1:1000 | Cell Signaling Technology | Polyclonal | 2101 |
| Rabbit anti-GAPDH | 1:1000 | Cell Signaling Technology | monoclonal | 5174 |

Antibodies for Flow cytometry

| **Primary Antibody** | **Dilution** | **Company** | **Clonality** | **Cat.No.** |
| --- | --- | --- | --- | --- |
| Mouse anti-ITG α5β1 | 1:200 | Merck Millipore | monoclonal | MAB1999 |
| Goat anti-ITG α5 | 1:200 | Santa Cruz | polyclonal | SC-6595 |
| Mouse anti-ITG β1 | 1:100 | Abcam | monoclonal | ab24693 |

| **Secondary Antibody** | **Dilutions** | **Company** | **Clonality** | **Cat. No.** |
| --- | --- | --- | --- | --- |
| Alexa Fluor® 488  (Donkey Anti-Goat IgG) | 1:500 | Abcam | Polyclonal | ab150129 |
| Alexa Fluor® 488 Conjugate  (Goat Anti-Rabbit IgG) | 1:500 | Cell Signaling Technology |  | 4412S |
| Alexa Fluor® 488 Conjugate  (Goat Anti-Mouse IgG) | 1:500 | Cell Signaling Technology |  | 4408S |
| IRDye 800CW Goat anti-mouse (H+L) | 1:10000 | LI-COR |  | 925-32210 |
| IRDye 800CW Goat anti-rabbit (H+L) | 1:10000 | LI-COR |  | 925-32211 |
