# Supplementary material for: Endothelial cell‐derived matrix promotes the metabolic functional maturation of hepatocyte via integrin‐Src signalling
Source: J Cell Mol Med. 2017 May 4;21(11):2809–22. doi: 10.1111/jcmm.13195 (PMC5661128; doi:10.1111/jcmm.13195)
Supplement: Supplementary file 8 — Table S2 Primers for Real‐Time RT‐PCR. [file JCMM-21-2809-s008.doc]

Table S2. Primers for Real-Time RT-PCR

| Accession number | Name | 5’-Sequence-3’ |
| --- | --- | --- |
| NM_000392.4 | ABCC2 | F:AGCAGGTATTCGTTGGTT  R:AGGTAGAGGCTTGGATTG |
| NM_000477.5 | ALB | F:TTGCCTTTGCTCAGTATCTT  R:ACAGCAGTCAGCCATTTCAC |
| NM_001077469.2 | CAR | F: CTGTCGGCAGAAGCCCTGGC  R: GCCCCCAGGAGTGTCCGGAT |
| NM_001875.4 | CPS1 | F:CTGACCCTGCCTACAAAG  R:CACCAGCAAACCTGAAAC |
| NM_000761.4 | CYP1A2 | F:AGTCTGTTCCCTTCTCGG  R:TGGCTCTGGTGGACTTTT |
| NM_000767.4 | CYP2B6 | F: AGGGAGATTGAACAGGTGATT  R:GATTGAAGGCGTCTGGTTT |
| NM_000771.3 | CYP2C9 | F: ACCCAAAGAACCTTGACACC  R: ACAGCAGCAGCCAGGCCATC |
| NM_000773.3 | CYP2E1 | F: CTGACCACCCTCCGGAACTAT  R: GGCCTTGGGTCTTCCTGAGT |
| NM_001202855.2 | CYP3A4 | F:GGCGGATGTTGAAGTGAG  R:GTTGGGTGTTGAGGATGG |
| NM_001935.3 | DPP4 | F: CATACAAATCACTGCTCCTG  R: GAATAGTTCTGAATCCTCCTG |
| NM_001306129.1 | FN1 | F:AAACTTGCATCTGGAGGCAAACCC  R: AGCTCTGATCAGCATGGACCACTT |
| NM_021784.4 | FOXA2 | F:CGCCCACTTCCAACTACCGC  R:GGCTCGTGCCCTTCCATCTT |
| NM_001287183.1 | HNF4A | F: TTTTGGCTACTTGAGTTGTG  R:CGTTCATTTCTGACCCTCT |
| NM_181501.1 | ITGA1 | F: AAATGGTTACCCTGTGCTGT  R: AATTGTGCCTCGTTTGAGAT |
| NM_002203.3 | ITGA2 | F: TATTCTGAGACTGCCAAGGT  R: GTTGCTGACAATAAAGGGTT |
| NM_002205.4 | ITGA5 | F: TGTGACTACTTTGCCGTGAACC  R: CGGAGATGAGGGACTGTAAACC |
| NM_002211.3 | ITGB1 | F: CTATCCCATTGACCTCTACTACCT  R: TTCCACAAATGAGCCAAATC |
| NM_000015.2 | NAT2 | F:CAGCCTAGTTCCTGGTTGCT  R:GGATCTGGTGCTCAAGAATG |
| NM_001018073.2 | PCK2 | F:TTCCCCACCGCACATACC  R:CCACCACCAATCCCAACG |
| NM_003889.3 | PXR | F: TGCGAGATCACCCGGAAGAC  R: ATGGGAGAAGGTAGTGTCAAAGG |
| NM_003060.3 | SLC22A5 | F:GTGAGGACGACTGGAAGG  R:CAAGGACAAACAGCACGAC |
| NM_005417.4 | SRC | F:TGGCTTCTGCTGTTGACTGG  R:CCTGGGAAGGTTGGAACTGAC |
|  | 18S | F:GTAACCCGTTGAACCCCATT  R:CCATCCAATCGGTAGTAGCG |
