# Supplementary material for: Endothelial cell‐derived matrix promotes the metabolic functional maturation of hepatocyte via integrin‐Src signalling
Source: J Cell Mol Med. 2017 May 4;21(11):2809–22. doi: 10.1111/jcmm.13195 (PMC5661128; doi:10.1111/jcmm.13195)
Supplement: Supplementary file 9 — Table S2 Primers for Real‐Time RT‐PCR. [file JCMM-21-2809-s009.docx]

**Supplemental figure Legends**

**Figure S1. Expression of ECM components in HUVECs.** Immunofluorescence staining for FN, collagen I, collagen IV, LN and CD31 in HUVECs. Scale bars, 75μm. Coll I, collagen I; Coll IV, collagen IV; FN, fibronectin; LN, laminin.

**Figure S2. The topography of different substrates.** The surface structure of the gelatin-treated coverslips were acquired using SEM, when they were cultured with endothelial cell growth medium containing 10%FBS for 72 hours (A), or HUVECs lysis (B). (C) The surface structure of the EC-matrix was acquired using SEM, when it was cultured with hepatocyte maintain medium containing 10%FBS for 72 hours. (D) The surface structure of the gelatin-treated coverslip was coated with FN was acquired using SEM. Scale bars, 2 μm.

**Figure S3. The properties of hASC-HLCs and human hepatocytes on different substrates.** (A) The morphology of hASC-HLCs cultured on the collagen I-coated substrate and EC-matrix for 72 hours. Scale bars, 100 μm. (B) Real-time RT-PCR analysis of hepatic markers in hASC-HLCs cultured on the EC-matrix and the collagen I-coated substrate. (C) Real-time RT-PCR analyses the expression of phase I and phase II drug metabolism enzymes, and drug transporters in human hepatocytes cultured on the EC-matrix and the collagen I-coated substrate. (D) Real-time RT-PCR analysis of CYP2C9 and CYP3A4 in hASC-HLCs cultured on the EC-matrix at different time points. The relative expression of each gene was normalized against 18S rRNA, and relative mRNA expression was presented with fold over the expression in the cells at day 1. Statistical significance compared to the mRNA expression in cells at day 1. *P<0.05, **P<0.01 (E) The basal activities of CYP2C9 and CYP3A4 were assessed in hASC-HLCs cultured on the EC-matrix and the Collagen I-coated substrate. RLU, relative luminescent unit.

**Figure S4. Efficiency of depletion of FN in HUVECs and the effect on hepatic maturation.** (A) Real-time RT-PCR analyses the FN mRNA levels in HUVECs 72 hours post-siRNA transfection. Statistical significance compared to the control siRNA, **P<0.01. (B) Immunofluorescence staining for FN was examined 72 hours post-siRNA transfection in FN siRNA treated- and control siRNA treated-EC-matrix. Scale bars, 75 μm. Quantitative analysis of FN was determined using Image J. Statistical significance compared to the control siRNA, **P<0.01. (C) Surface views of the FN depleted EC-matrix and the control EC-matrix were acquired using SEM. Scale bars, 2 μm. (D) Real-time RT-PCR analyses the expression of metabolic genes in hASC-HLCs cultured on different substrates. (E) The basal activities of CYP2C9 and CYP3A4 were assessed in hASC-HLCs cultured on the FN depleted EC-matrix and the control EC-matrix. (F) The activities of CYP2C9 and CYP3A4 after induced by rifampicin were assessed in hASC-HLCs cultured on different substrates. Statistical significance compared to the EC-matrix, **P<0.01. control, the control EC-matrix；FN, fibronectin; FN depleted, the FN depleted EC-matrix; RLU, relative luminescent unit.

**Figure S5. Efficiency of depletion of α5 integrin in hASC-HLCs and the effect on hepatic metabolic maturation.** (A) Real-time RT-PCR analysis of the α5 integrin mRNA levels in hASC-HLCs 72 hours post-siRNA transfection. (B) Immunofluorescence staining for α5β1 integrin was examined 72 hours post-siRNA transfection in hASC-HLCs. Scale bars, 75 μm. Quantitative analysis of α5β1 integrin was determined using Image J. Statistical significance compared to the control siRNA, **P<0.01. (C) The basal activities of CYP2C9 and CYP3A4 were assessed in hASC-HLCs-integrin α5 siRNA and hASC-HLCs-control siRNA cultured on EC-matrix for 72 hours. ITG, integrin; RLU, relative luminescent unit.

**Figure S6. Efficiency of depletion of Src in hASC-HLCs.** (A) Real-time RT-PCR analysis of the Src mRNA levels in hASC-HLCs cultured on the EC-matrix 72 hours post-siRNA transfection. (B) The protein level of Src was examined by western blot. Statistical significance compared to the control siRNA, **P<0.01.
